# Comparing the Effect of Product-Based Metrics on the Translation Process

**DOI:** 10.3389/fpsyg.2021.681945

**Published:** 2021-08-16

**Authors:** Bram Vanroy, Moritz Schaeffer, Lieve Macken

**Affiliations:** ^1^LT^3^, Translation, Interpreting and Communication, Ghent University, Ghent, Belgium; ^2^TRA&CO, Translation Studies, Linguistics and Cultural Studies, Johannes Gutenberg University, Mainz, Germany

**Keywords:** translation studies, translation difficulty, eye tracking, syntax and grammar, translation process and product, translation process research, lexicosemantics

## Abstract

Characteristics of the translation product are often used in translation process research as predictors for cognitive load, and by extension translation difficulty. In the last decade, user-activity information such as eye-tracking data has been increasingly employed as an experimental tool for that purpose. In this paper, we take a similar approach. We look for significant effects that different predictors may have on three different eye-tracking measures: First Fixation Duration (duration of first fixation on a token), Eye-Key Span (duration between first fixation on a token and the first keystroke contributing to its translation), and Total Reading Time on source tokens (sum of fixations on a token). As predictors we make use of a set of established metrics involving (lexico)semantics and word order, while also investigating the effect of more recent ones concerning syntax, semantics or both. Our results show a, particularly late, positive effect of many of the proposed predictors, suggesting that both fine-grained metrics of syntactic phenomena (such as word reordering) as well as coarse-grained ones (encapsulating both syntactic and semantic information) contribute to translation difficulties. The effect on especially late measures may indicate that the linguistic phenomena that our metrics capture (e.g., word reordering) are resolved in later stages during cognitive processing such as problem-solving and revision.

## 1. Introduction

Translation difficulty prediction, which aims to assess the difficulty of a translation task, is a topic of interest within Translation Studies that can benefit both pedagogical and research settings. Advances in translatability could for instance ensure that appropriate text material is used in translation classes, and to create general-purpose machine translation (MT) systems that are trained on a balanced mix of simple and hard texts. On the other hand, it could also help the research fields of Translation Studies and psycholinguistics to select source material of suitable translation difficulty for experiments. Even though a well-established methodology to quantify a source text's translatability does not exist (yet), the problem of translation difficulty has gained some attention over the years.

The PreDicT project^1^ (Predicting Difficulty in Translation) aims to contribute to the field of translatability by investigating source text language features that add to a text's translation difficulty.^1^ As described above, the application of advances in this field could be to predict the translation difficulty of a source text, or parts of it, without having access to a translation. That would allow users to automatically rate a text or highlight its difficulties without the need of translating it beforehand. The PreDicT project has particularly focused on syntactic similarity and divergence between a source text and its translation. In previous work (Vanroy et al., 2019), two metrics were introduced to calculate the word and word group movement on the sentence level. In addition, a machine learning system was built that could predict these word and word group reordering values by only using source text information with a moderate Pearson *r* correlation. Additional sentence-level metrics were introduced in Vanroy et al. (2021). In the current paper, however, we take a more fine-grained approach and make these metrics available on the word level so that meaningful translation process analyses can be done to investigate their impact on the translation task.

We examine the effect of a number of predictor variables on translation process data as a proxy for cognitive effort and, hence, difficulty, as is usual in translation process research (Muñoz Martín, 2012). We include metrics that are intended to measure syntactic or (lexico)semantic (dis)similarities between a source text (ST) and its target text (TT), or both. Some metrics require multiple translations (and are entropy-based), and others can be calculated on single translations. The unit of interest is the word, but some of the metrics are calculated with word group information in mind. The goal of this paper is not to create a single model with the highest predictive power but to make a comparison between the predictive capabilities of metrics that differ along a number of dimensions: syntactic vs. lexicosemantic ones, based on different units (words vs. word groups), and those relying on multiple translations (entropy) vs. on a single translation. The current research can thus serve as a peek into the effects that such different metrics have on process data as a proxy for translation difficulty. We test their effect on three different eye-tracking measures on the source tokens (section 3.3), both early and late. The early measure that we look into is First Fixation Duration (FFDur; duration of first fixation on a token). The late eye-tracking measures are Eye-Key Span (EKS; duration between the first fixation on a source token and the first keystroke to produce its translation; Dragsted and Hansen, 2008; Dragsted, 2010) and Total Reading Time on source tokens (TrtS; sum of all fixations on a token). We only focus on the Total Reading Time on source tokens so, for brevity, “TrtS” is also referred to as Total Reading Time in the remainder of this paper.

Results of the current study can be used in the grander scope of a translatability system in future work. If we find that our predictors indeed affect translation difficulty, then they can be modeled (predicted) by only making use of the source text, similar to our previous work (Vanroy et al., 2019). Such predictions may then serve as input features for a translation difficulty prediction system.

This paper is structured as follows. First an overview of related research regarding literal translation, the relationship between ST and TT and how to quantify it, and the translation unit is discussed. Then, the experimental set-up is described in section 3, with specific attention for the data and model description. Section 4 reports the results, which are elaborated on in the discussion (section 5). Finally, we end with broad conclusions and suggestions for future research in section 6.

## 2. Related Research

A lot of work has been done on the relationship between ST and TT, particularly on the concept of literal translation and the (formal) transfer of the source text to the target. We will discuss one specific way how literal translation can be operationalised (Schaeffer and Carl, 2014), which leads us to different ways of how the relationship between a source and target text can be measured (section 2.2). This section is extensive because many of the measures to quantify the relationship between ST and TT that it describes will also be used as predictors in our experiments. Finally, research concerning the unit of translation is described, as it relates to our decision to include predictors that are calculated based on word as well as on word group information.

### 2.1. Literal Translation

“Literal translation” is often contrasted with free translation and yet a single definition is not available (Shuttleworth and Cowie, 2014, p. 95–97). The concept has been used in different ways to mean different things (see Halverson, 2015, for an extensive overview of varying interpretations). For instance, some consider literal translation ungrammatical and outside the acceptable norm depending on the genre. In such a view, literal translation is considered as nothing more than what Seleskovitch (1976) calls code switching, the technical conversion of one symbol to another. Others restrict literal translation to mean word-for-word translation that leads to a necessarily “grammatically and idiomatically correct TL [target language] text” (Vinay and Darbelnet, 1995, p. 33), or go even so far that the only requirement for literal translation is that the translation is “structurally and semantically *modeled upon* the ST fragment while respecting TL grammatical constraints” (Englund Dimitrova, 2005, p. 53; our emphasis).

Abstracting away from the discussion above, and without defining literal translation itself, Chesterman (2011, p. 26) refers to the *literal translation hypothesis* that states that “during the translation process, translators tend to proceed from more literal versions to less literal ones.” He does not make any claims about what the starting point is nor about what a “most” and “least” literal translation would look like. The literal translation hypothesis simply states that initially formal features of the source text have a large effect on the (perhaps mental or “interim”) translation that is being produced and that this effect decreases over the duration of the translation process. The literal translation hypothesis has received supporting evidence from translation process studies that measure the effects of literality metrics (see below) on process data (e.g., Bangalore et al., 2015, 2016; Schaeffer et al., 2016b). Such experiments show that the translation procedure starts from a more literal translation, but when this is not possible due to the constraints of TL or other contextual or extralinguistic factors, non-literality must inevitably increase, which—the experiments show—goes hand in hand with a higher requirement of cognitive effort. These findings also (implicitly) support the (revised) Monitor Model (Tirkkonen-Condit, 2005) that suggests that literal translation is the “default rendering procedure” (p. 407–408). The translation process is monitored by an internal monitor function and when it encounters an issue in the rendered translation (e.g., contextual or grammatical), it intervenes and other, less literal, approaches are considered.

Schaeffer and Carl (2013) introduce a revised, recursive, version of the Monitor Model. It suggests that default (literal) translations are produced based on the shared representations of source language (SL) and target language (TL) items that are active in the mind of the translator. If the monitor recognises that the influence of the source text leads to unacceptable (literal) target text, then the automatic process is interrupted. Similarly, Carl and Dragsted (2012) propose that understanding the source text and producing a translation occur in parallel. The production process is monitored and when issues arise, alternative translation options are considered. Such parallel processing is especially straightforward in a copy task but also in literal translation empirical evidence is found to support this view.

In an effort to define literal translation in terms of the similarity between the source and target text, Schaeffer and Carl (2014, p. 29–30) propose that three criteria need to be met:

1. The word order is identical in the ST and TT;
2. ST and TT items are one-to-one translation equivalents;
3. Each ST word has only one possible translated form in a given context.

These criteria for literality have served as the starting incentive for the creation of similarity metrics that compare the syntactic and (lexico)semantic properties of a source sentence with its translation. As such, these metrics operationalise literality and can be used to measure the impact of literality, but also of divergent structures in general, on the translation process.

### 2.2. Measuring the Relationship Between ST and TT

The literal translation hypothesis and the way that is has been operationalised, is often used in translation process research as predictors for cognitive load during translation (Muñoz Martín, 2012). A high cognitive load is indicative of difficulties that a participant is experiencing. Reichle et al. (2009) show that during reading, a participant processes previous information while absorbing new text and during this stage of postlexical processing lexical, semantic or syntactic difficulties may arise that involve previously encountered words. These difficulties require attention on the word that triggered the problem or regressions to previous information to solve them. Hence, measures involving eye tracking, keyboard logging, and duration data can provide hints toward the cause of translation difficulties because they indicate where and for how long a translator is paying attention.

Although translation difficulty can be approached from different angles, for instance by looking at extra-linguistic properties or only at the source text, a particular set of translation difficulties deals with resolving the relationship (and similarities or differences) between the source text and a plausible translation. We focus on the latter type of difficulties. The metrics that follow were all suggested in previous work to model the relationship between a source sentence and its translation in different ways. They all rely on word alignment information. Word alignment is the linking of a source word with its translated word(s) so that the relationship between smaller units can be quantified.

#### 2.2.1. Cross

**Figure 1:**
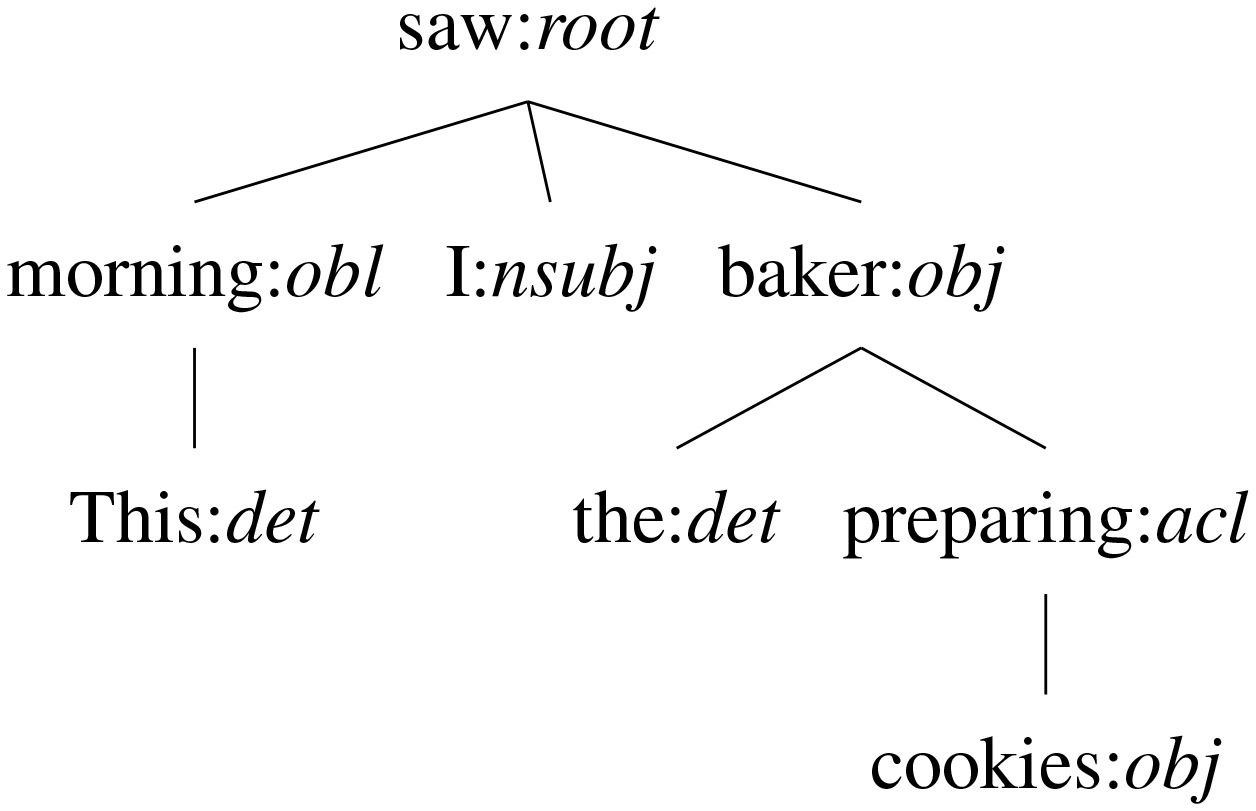
Example dependency tree of the sentence “This morning I saw the baker preparing cookies”.

To be able to investigate the first point of the definition of literal translation of Schaeffer and Carl (2014) “the word order is identical in the ST and TT,” the authors suggest a metric that can quantify word reordering. Cross (Schaeffer and Carl, 2014; Carl et al., 2016; Carl and Schaeffer, 2017) quantifies the reordering of a word's translation relative to the position of the previous word's translation. That means that Cross values can be positive (when the translation is placed after the previous one) or negative (when it is placed before the previous translation). In an absolute literal translation where a one-to-one relation exists between every source word and a corresponding target word (Schaeffer and Carl, 2014), and where the word order is maintained, every word has a Cross value of 1 (because each translation is one step further than the previously translated word). An example of Cross is given later on in Figure 1 where it is compared with other reordering metrics. In previous research, (absolute) Cross values were found to have a significant positive effect on First Fixation Duration and Total Reading Time on source tokens (Schaeffer et al., 2016b).

#### 2.2.2. HCross

As an extension to the relative word reordering of a single translation, Schaeffer and Carl (2017) introduce the concept of HCross, which is an entropy-based variant of Cross. Entropy (Formula 1) is a measure from information theory to quantify the added value of new information (Shannon, 1948). Applied to our use cases in Translation Studies, entropy can be interpreted as the amount of agreement between translators or the amount of uncertainty with respect to a given phenomenon. Low entropy values mean high agreement (or low uncertainty), and high entropy would indicate low agreement (high uncertainty). As such, multiple translations of the same text need to be available to have meaningful entropy results. By taking as many shadow translations into account as possible (“possible alternative translations defined by the systemic potential of the target language;” Matthiessen, 2001, p. 83), the hope is to approximate all translation possibilities and by extension model the entropy; the uncertainty for choosing between all those options. It has been suggested that approximately ten translations are needed when calculating entropy to achieve a Pearson correlation of more than *r* = 0.8 with the real population (Carl, in press) although that finding was restricted to a semantic metric called word translation entropy (HTra), which will be explained next.

(1) $$H(X)=-\sum_{event\in X}P(event)log_{2}P(event)$$

**Table:** where:

*X*	a set of possible unique events
*P(event)*	the probability of a given event

The general entropy formula is applied to Cross by the authors as in Formula 2. Instead of only considering a single translation, entropy is calculated on all available translations of the same source text. In other words, by taking into account the translations of the same source text by different translators, HCross can quantify how pre-determined the reordering of a source word must be. If there is little variation in the Cross values for a source word among different translators, then the entropy will be low. For high variance, the entropy value will be high. Put differently, if translators reorder a source word in the same way (and agree about the repositioning of the translation), then HCross will be low, and otherwise it will be high. Schaeffer and Carl (2017) find that HCross has an effect on the duration of the Eye-Key Span. That suggest that more disagreement about word reordering has an effect on EKS, possibly indicating that when participants have many possible word orders to chose from, the decision-making process takes more time.

(2) $$HCross(w,C)=-\sum_{c\in C}P(c|w)log_{2}P(c|w)$$

**Table:** where:

*C*	a set of unique Cross values associated with w in this context
*P(c|w)*	the probability that w has a Cross value of c in this context

#### 2.2.3. Word Translation Entropy (HTra)

Where HCross is a way to quantify the uncertainty of word reordering, word translation entropy (HTra; Carl and Schaeffer, 2014; Carl et al., 2016) does the same for the lexical choice for a translation. For a given source word, HTra takes all translations of that word in the specific context into consideration. Depending on how much agreement or disagreement there is between translators to choose the same target word, HTra will be low or high, respectively. Applying Formula 1 to word translation entropy, HTra can be defined as Formula 3.

(3) $$HTra(w,T)=-\sum_{t\in T}P(t|w)log_{2}P(t|w)$$

**Table:** where:

*T*	a set of unique translations of w in this context
*P(t|w)*	the probability that w is translated as t in this context

This measure is thus a way to see how many translations (lexical entries) are suitable translations. It gives us a (limited) insight in the different options that translators can choose from (contextual lexicon). A high HTra value means that many options are available and that a single, straightforward choice is not necessarily available. As a consequence, a high word translation entropy is expected to have an impact on process data as well: more choices to choose from for a given word in a specific context, is likely to require more time to make a decision. This has been confirmed in a number of studies. Effects of HTra were reported on total production duration (Carl and Schaeffer, 2017), First Fixation Duration and Total Reading Time on source tokens (Schaeffer et al., 2016b), and Eye-Key Span (Schaeffer and Carl, 2017). This would mean that the effect of word translation entropy is present in both early and late processing stages during translation. HTra has been shown to correlate with HCross, both within and across languages (Carl et al., 2019; Carl, in press). That is unsurprising: different words in the target language may require different word orders, which in turn may be an indicator of different syntactic structures.

#### 2.2.4. Joint Source-Target Alignment/Translation Distortion Entropy (HSTC)

Recently, a new entropy-based metric has been introduced that incorporates different types of information into a single metric (Carl, in press). It is called “joint source-target alignment / translation distortion entropy,” or HSTC for short, and takes into account translation and reordering probabilities. Specifically, a given source word *w* is part of a group of source words *s*, which is aligned to a group of target words *t*. An alignment group is defined as a number of source and target words that are aligned with each other. These groups represent meaning-equivalent expressions in the context of the sentence. All words in a source group *s* have the same Cross value *c*. As such, the joint alignment/distortion probability for a given source word *w* is based on its associated source group *s*, the alignment with target group *t*, and the corresponding Cross value *c*. These probabilities can then be used to calculate the entropy (Formula 4). In a way, HSTC encompasses both HTra and HCross discussed above. It is intended as a single metric to measure the (non-)literality of a translation, both (lexico)semantically and syntactically.

(4) $$HSTC(w,A)=-\sum_{(s,t,c)\in A}P(s,t,c|w)log_{2}P(s,t,c|w)$$

**Table:** where:

*w*	a given source word
*A*	a set of unique triplets of associated values of w in this context
*s*	the source word group that w belongs to in this set, aligned with its respective *t*
*t*	the target word group that w belongs to in this set, aligned with its respective *s*
*c*	the Cross value of all words in group *s*
*P(s,t,c|w)*	the probability that *w* is associated with this source group *s*, target group *t*, and Cross value *c* in this context

Carl (in press) shows that, perhaps unsurprisingly, HSTC correlates strongly with both HTra and HCross, which implies that uncertainty in choice of lexical translation goes hand in hand with similar uncertainty about the reordering. Similar to the aforementioned measures, Carl (in press) presents significant effects of HSTC on production duration during translation.

With the exception of Cross, the above measures are all meant to be calculated involving a relatively high number of translations. The main idea is that a sufficient number of translations approximate all the possible choices that translators are faced with, and that more choices (or less-straightforward ones) lead to a more difficult translation process. Vanroy and colleagues introduced different syntactic metrics that are not reliant on multiple translations and each focus on different aspects of syntactic differences between a source text and its translation (Vanroy et al., 2019, 2021). Instead of trying to comprise “one metric to rule them all” such as HSTC, where a lot of information is included in a single measure, they split up syntactic (dis)similarities between a source and target text into individual measures.

#### 2.2.5. word_cross

Cross, as discussed above, is a metric to measure the reordering of a word's translation relative to the translation of the previous word. It is directional, in the sense that a word and its translation can have different values. In Vanroy et al. (2019), we suggest a different approach to word reordering that is bidirectional and absolute. We will call this metric word_cross in the current paper to distinguish it from the aforementioned Cross value (Carl et al., 2016; Carl and Schaeffer, 2017). First, word_cross is calculated as the number of times an alignment link of a specific word crosses the alignment link of *any* other word in the sentence. Formally, two alignment links cross each other if the order of the source words is inverted on the target side.

**Figure 2:**
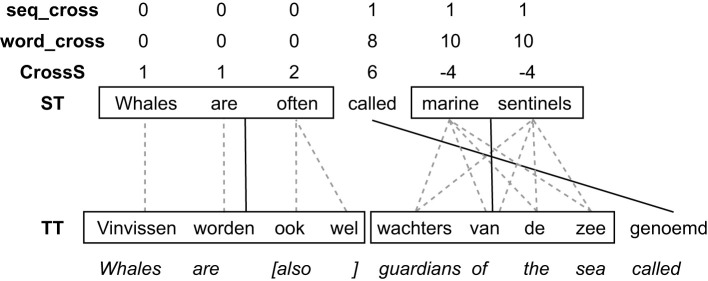
A visualisation of Cross, word_cross and sequence cross in Example 2.

In other words, whereas a word's Cross value is determined by the reordering of its translation relative to the previous word's translation, its word_cross value is impacted by the reordering of *all* words in the sentence, including its own. The implication of this is that the cross value of a target word is the same as the cross value of its aligned source, at least in one-to-one alignments. If a word is aligned with multiple target words, we can choose to take the average cross value of its alignments, or sum them up (in this paper we sum them), which means that for some aligned structures the cross value of a source word could differ from its aligned target word, because that target word is aligned with other source words as well. In Vanroy et al. (2019) and later in Vanroy et al. (2021), this metric was only available as an aggregated value on the sentence level and could therefore not be used for word-level predictions or correlations. The reason for this is that we initially wanted to make word (group) order distortion predictions for a given sentence, i.e., we were answering the question whether we can predict the difference in word (group) order between a source sentence and its translation. Figure 2 illustrates the difference between word_cross and other reordering metrics in a following section.

#### 2.2.6. seq_cross

Similar to Gile (1995, pp. 101–102), we consider that the translation unit can vary and is not necessarily restricted to only words nor to only word groups. The unit of translation may differ between translators, between tasks and even specific texts and difficulties (section 2.3). Therefore, we also investigate the effect of word *group* (or *sequence*) reordering on process data. Similar to word_cross above, sequence cross (or seq_cross) was introduced in Vanroy et al. (2019) and further discussed in Vanroy et al. (2021). Word groups can be created based on the alignments of the involved words and restrictions apply as per the requirements in Requirement 1, taken from Vanroy et al. (2019). If a word does not belong to a group that follows these requirements, then that word's original word alignment will be used as a “singleton” sequence alignment as well.


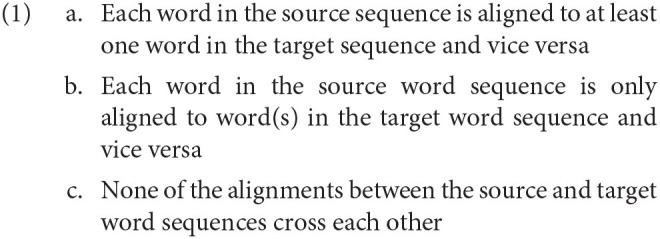


So looking at this from a technical perspective, aligned word groups are created as described above, and for these word groups and their alignment a cross value is calculated in the same fashion as for word_cross. It is ensured that these groups are as large as possible according to the requirements. In section 3.1, an illustration of seq_cross is given as comparison to the aforementioned reordering metrics (Figure 2).

#### 2.2.7. Aligned Syntactic Tree Edit Distance (ASTrED)

In Vanroy et al. (2021) we finally also introduced a metric that we call Aligned Syntactic Tree Edit Distance (ASTrED) that compares the linguistic structure of a source and target sentence. The syntactic structure of a sentence can be represented as a hierarchical tree where each child is a lower item in the tree to its parent. Specifically, we make use of dependency trees where each word has a to-relationship with its parent in the tree. That means that each node in a tree is the dependency role label of that word (for instance, a word can have the role of subject *to* the root verb; see Figure 1 for an example). The structure of the source tree can then be compared with a target tree representation to find structural differences between the two. To do so, however, the label set and way of structuring a sentence needs to be comparable between languages in the first place. Therefore, we make use of the Universal Dependencies annotation scheme^2^ (UD), which is an initiative to facilitate and accelerate multilingual, comparable research (Nivre et al., 2016). It is specifically designed to do away with the prior difficulty of comparing two languages syntactically. As an example, Figure 1 shows the dependency tree of the sentence “This morning I saw the baker preparing cookies” where the nodes are represented as word:dependency-label. In reality, however, only the dependency label is used in comparing the structures.

Because we can be certain that the structures of a source text and its translation use the same annotation scheme, we can compare the tree representation of a source sentence and its translation. One could naively measure the tree edit distance (TED) between the two, a common metric to measure differences between trees. TED looks for the most optimal way to transform the source tree into the target tree by making use of different operations: match (when a source node has the same label^3^ as a node on the target side in the same position), insertion (when a node is not present in the source tree but needs to be inserted in the target tree), deletion (when a source label is not present in the target tree and needs to be deleted) and substitution (also called rename; when a source label is structurally correct but its label needs to be changed to be identical to a target node). Every operation has a cost attached to it, and the TED algorithm needs to look for the sequence of operations that has the lowest total cost. In our case, match has no cost to it (and is thus the preferred operation if possible), and the others have a cost of 1.

TED as-is is a naive approach, however, as it will not take word alignments into account. It will simply find the most optimal solution to change the source sentence *structure* into the target structure, irrespective of word alignments and effectively ignoring any semantic or structural correspondence between the source and target sentences. ASTrED, on the other hand, can be seen as a preprocessing procedure for syntactic trees that ensures that only aligned words can match in the source and target tree by merging the node labels in both the source and target tree to include information about the aligned words. This procedure is described in much detail in Vanroy et al. (2021) and will not be duplicated here for brevity's sake. Important to know is that ASTrED changes the node labels in such a manner that the nodes of aligned source and target words will end up having the same label in their respective trees. After this preprocessing step, TED can be calculated. Because match is a preferred operation (cost 0), this ensures that TED will try to match aligned words (rather than words that coincidentally have the same label) in the tree and fill out the rest of the tree with substitution, insertion, and deletion operations.

### 2.3. Unit of Translation

In Translation Studies, (the size of) the unit of translation remains a much discussed topic, approached from different directions. A distinction can be made based on the focus of the research, i.e., the translation process or its product. In product-emphasised studies, it is generally accepted that the translation unit (TU) is a *pair* of (a) source item(s) and its corresponding target item(s). In process-based studies, the focus lies on the source text. The translation unit here is considered to be the source item(s) that a translator processes one at a time (Malmkjær, 2006). An overview of this dichotomy is given in Alves and Vale (2009). In this paper we are particularly interested in the translation unit in the first interpretation because we compare the source text with its translation (the product). However, a lot of work has been done on the unit of translation during the translation process. For instance, Dragsted (2005) found that the size of translation units (or “segments”) differs depending on the difficulty level of a text (smaller units for difficult text) and between novice and professional translators. Professionals tend to work on larger chunks of text at a time. Translation units, in the work of Dragsted (2005) but also in related research, are frequently defined as the productive part of the process in between two pauses of a specified length where keyboard activity can be observed. In the experiment of Dragsted (2005), this pause length was chosen by using a formula that takes idiosyncrasies of translators into account.

Rather than investigating a single type of translation unit in process data, Carl and Kay (2011) proposes the usage of different kinds of units as proxies for the TU itself. Source and target pairs of items can be segmented into alignment units (AU; aligned source and target words), the eye-tracking data in fixation units (FUs; consecutive fixations segmented by a pause of a given threshold), and the keystroke data in text production units (PUs; coherent typing behaviour segmented by a pause). By separating the concept of a unit across different parts of the translation process, the authors intend to approximate the “properties and shapes of Translation Units” (p. 972). When the boundaries that constitute these units are chosen correctly, PUs are shown to be a rough approximation of the translation unit, i.e., a unit of cognitive activity. The size of these units in terms of time, as segmented by pauses, differs between novices and professional translators. The PUs of professionals are larger, which indicates the processing of larger chunks at a time, which lends to support to the findings by Dragsted (2005). By extension, Carl et al. (2016) suggest activity units (CUs). Activity units can be categorised according to the activity type at hand such as “translation typing while reading the source text” or “target text reading.” There are eight types in total (Carl et al., 2016, p. 38–39).

Alves and Vale (2009), and continued in later work (Alves et al., 2010), make the distinction between micro and macro translation units. A macro TU encapsulates a series of micro TUs. A micro TU is therefore more similar to the TU as it was discussed up to now (a unit of activity segmented by a pause of a given length). Macro TUs, on the other hand, are collections of micro units that are all related to the same source segment. In other words, when different micro TUs all contribute to the production of the translation of a specific word (by inserting or deleting characters or by revising previously produced text), then all of those together are considered the macro TU.

Immonen and Mäkisalo (2010) aim to find overlaps and correlations between syntactic units and the pause boundaries that are typically used to segment translation units. Among other things, their results show that in translation the processing of small units require more processing time compared to a monolingual task, and larger linguistic units are relatively speaking less time demanding. Their explanation for this is that during translation a translator spends a lot of time on getting the translation of small units right in terms of its similarity to the source text. But for larger linguistic structure this integration requires less time because they are easier to copy from the source text (e.g., the internal structure of a text or paragraph). These findings are confirmed in a later study as well (Immonen, 2011).

It is clear that research is actively involved in the translation unit, but clear-cut definitions do not exist. A translation unit is a variable concept: it differs between participants and tasks, and may or may not necessarily correspond to syntactic units. In this paper, however, we rely on the minimal product-based view that a translation unit is a pair of (aligned) source and target items. We investigate both small, word-based units and larger (word group) units.

## 3. Materials and Methods

In this section, we first discuss a couple of improvements that were made for the current paper to metrics that we introduced in earlier work. Then, we describe our dataset and the processing that was applied to it, followed by a description of the regression models that were built and the involved variables.

### 3.1. Improvements of Existing Metrics

In section 2.2, we discussed methods to quantify the relationship between a source sentence and its translation. Methodologically, the current paper makes some small improvements to the metrics that we introduced in Vanroy et al. (2021).

First and foremost, previous work focuses on sentences. In the current study we zoom in on individual words. That means that some metrics were re-implemented so that word-level analyses could be done.^4^ This is particularly the case for word_cross, seq_cross, and ASTrED. The sequence cross value of a group is passed on to all the words belonging to that group. Each word thus has a word_cross value, based on word alignment and its own reordering, and a seq_cross value that is based on the alignment of the word group that it belongs to. These sequence alignments (alignment between two word groups) can greatly reduce the number of alignments and, consequently, the cross values calculated on these groups (seq_cross) can be much smaller than their word_cross equivalent because there are less (group) alignments present in the sentence to cross compared to word alignments.

seq_cross itself was improved as well. We now consider m-to-n alignments of consecutive items as valid aligned word groups. In other words, Requirement 1c does not apply to these so-called multi-word groups (MWGs), but as an alternative requirement all source words need to be aligned with all target words of the construction. The assumption here is that m-to-n alignments are used for groups of words or phrases that cannot be easily compositionally aligned, such as idioms or free translations of specific concepts. Semantically, however, the source and target side should constitute the same concept or phrase. Note that this does not necessarily mean that from a monolingual perspective these constructions are multi-word expressions or idiomatic expressions: MWGs are purely based on the alignments between the source and target words belonging to the construction. As an example of a MWG, consider the following translation, where “marine sentinels” — “wachters van de zee” constitutes a MWG according to our specification and as such only one alignment link will be needed between the two groups rather than the m-to-n word alignments (which would lead to a lot of crosses because all word alignments in m-to-n alignment cross each other).


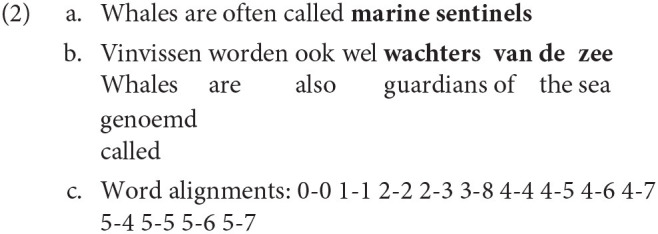


Note that allowing m-to-n alignments to be groups, also greatly reduces the sequence cross value of other words: because “called” is aligned with “genoemd” it crosses the m-to-n alignment, leading to a large word_cross value of 8. However, its sequence alignment (which is the same as its word alignment), has a seq_cross value of 1 because the m-to-n construction that it crosses is considered a valid sequence and only has one alignment link connecting “marine sentinels” to “wachters van de zee” instead of eight. Example 2 can be visualised as in Figure 2. It shows the differences between Cross, word_cross, and sequence cross. The groups of words that adhere to the requirements above are boxed in and aligned (solid black lines). Their original word alignments are given in grey dotted lines. If a word does not belong to a multi-word group, it is its own singleton group (like “called” in the example). Cross and word_cross are calculated on the alignments of the single words, whereas sequence cross uses the alignments between word groups. On the word-level (based on word alignments), “called” crosses eight alignment links. On the word-group level, however, this is reduced to only one.

Minimal changes were made to ASTrED as well to accommodate the focus on the word level. For each source word we check whether it was matched (and not changed) or whether an edit operation was necessary to transform this specific node to create the target tree (changed). These operations can only be deletion or substitution because insertion can only happen for target words. Each word, then, has an astred_change value of “FALSE” (match) or “TRUE” (no match), indicating whether a specific operation needs to occur on this word.

### 3.2. Data and Processing

For our experiments, we use a subset of ROBOT (Daems, 2016), a pre-existing English-to-Dutch translation process data set containing translations and post-edits of MT of eight different texts. In terms of complexity and readability, the authors chose all texts to be comparable by means of Lexile scores and readability formulas (section 4.1.1 Daems, 2016). Lexile scores are a standard measure for text complexity and comprehension levels.^5^ Each participant was asked to post-edit machine translations of four texts and translate the remaining four from-scratch. In the current paper we only make use of the from-scratch translations. The translation process was recorded using an EyeLink 1000 eye tracker in combination with Inputlog (Leijten and Van Waes, 2013) and CASMACAT (Alabau et al., 2013). Participants were allowed to make use of external resources and such information was captured with Inputlog. The translation process itself, that is the time when a translator was inside the CASMACAT environment reading (fixating) and translating (typing), was recorded with CASMACAT. These two types of data were then combined programmatically (section 4.5, Daems, 2016). This process ensures that eye-tracking information is only recorded inside CASMACAT while a participant is translating. It also makes sure that the final dataset contains information that is relevant to the tool (CASMACAT or external) that was being used at a given moment. After the translation process was completed, the final translations were manually sentence and word aligned with the source texts with YAWAT (Germann, 2008).

The full dataset consists of post-edited and from-scratch translations of eight news articles by ten student translators (P1-P10) and twelve professionals (P21-P32;P34^6^). Because the translations of P10 were not aligned, and because our metrics require word alignments we could not include that participant's data. P32's eye-tracking data was not included because of its poor quality, probably due to contact lenses. The product information of P32 was taken into account for the calculation of entropy values, however. In total, that leaves us with 21 translators who each translated three or four texts. That means that the eight texts each have between nine and eleven translations. Segments that were not translated as exactly one target sentence were not included because one of our metrics requires a linguistic parse tree, which is generated on a per sentence basis.

The translation process research database (Carl et al., 2016, TPR-DB)^7^ was used to generate useful overview tables based on the collected data. Relevant process features were automatically calculated by the TPR-DB, including fixation durations and keystroke information. Product features, such as the (H)Cross feature (Schaeffer and Carl, 2014, 2017), are derived from the final translation and its relation to the source text and are added automatically as well. All this information can then be exported into so-called TPR-DB tables where each word is supplied with all of the aforementioned measures and more.

The metrics proposed by Vanroy et al. (2019, 2021) were added at a later stage. A Python script that we provide in our library^8^ can calculate and add the metrics automatically to the TPR-DB tables. To create the linguistic structures that are needed for one of our metrics, we rely on stanza (version 1.2) (Qi et al., 2020) to parse both source and target sentences into the Universal Dependency schema (Nivre et al., 2016) (version 2.7).

### 3.3. Regression Models

We built regression models with dependent variables First Fixation Duration (FFDur), Eye-Key Span (EKS), and Total Reading Time on source tokens (TrtS). FFDur, a very early measure, is the time in milliseconds of the first fixation when a source word is first encountered. Eye-Key Span is the time between the first fixation on a source word and the first keystroke that contributes to the translation of that word (EKS; Dragsted and Hansen, 2008; Dragsted, 2010). It is therefore a relatively late measure because when a translator starts typing the target word, it is assumed that they have at least processed the source word and perhaps some of its context sufficiently to start producing a translation for it. TrtS, finally, is the total time (sum of fixations) that a translator has spent looking at a source word, irrespective of when fixations occurred. It is therefore a very late measure. Initial models for First Pass Duration (FPD) and Regression Path Duration (RPD) were created but those did not yield promising results and were not included in the final paper. FPD is the sum of the first consecutive fixations on a word before moving to any other word (before or after the current word). RPD is a late measure that is the sum of all fixations on a word including regressions to previous words before a fixation to the right of the current word is registered.

We use metrics that have been discussed in detail before (section 2.2) as predictors in our regression models. We repeat them below for clarity. These predictors were chosen because there is a lot of variation in the aspects that they model: some are semantic, others are syntactic; some require multiple translations and others do not; some are word-based whereas others make use of word groups. Our experiments compare these different aspects to one another in terms of their effect on the translation process.

- Cross (section 2.2.1): relative word reordering. We use the absolute value of Cross in our experiments (Schaeffer et al., 2016b; Carl and Schaeffer, 2017; Schaeffer and Carl, 2017)
- HCross (section 2.2.2): entropy version of Cross
- HTra (section 2.2.3): word translation entropy
- word_cross (section 2.2.5): absolute word reordering
- seq_cross (sections 2.2.6, 3.1): absolute word group reordering
- astred_change (sections 2.2.7, 3.1): compares linguistic structure of source and target sentence while taking word alignment information into account

For our analyses, we used R (R Core Team, 2020) and the package lme4 (Bates et al., 2015) for linear mixed regressions. To test for statistical significance of the effects, we made us of the R package lmerTest (Kuznetsova et al., 2017). We used the MuMIn package (Bartoń, 2009) for calculating *R*^2^ for fitted models. Model comparison was carried out with the anova function from the base stats package. Multicollinearity was assessed by using the vif.mer() function (Frank, 2011). In order to assess whether the normality assumption of model residuals was met we used the package moments (Komsta and Novomestky, 2015) to compute kurtosis and skewness of model residuals. A skewness of >|2| and kurtosis of >|7| are considered as severe deviations from the normality assumption regarding model residuals (Kim, 2013). We use the effects package (Fox, 2003) to visualise results of models without residual outliers.

Prior to model building, for each dependent variable, we excluded data points from the raw data which differed by more than 2.5 standard deviations from the mean for each participant. This resulted in no case in a loss of more than 3%. All models had, as random variables, participant and item (this was the source word for all models). The first model we built always included HCross—whether it was significant or not. We then included word form frequency (from the English Lexicon Project; Balota et al., 2007) for the reading times on the source text. We also included the sequential numbering of tokens in the source texts (STid; source text ID) and in the sentence (word_id; word ID) as predictors. However, if inclusion of both these variables meant that the model did not converge, only one of these—whichever was more significant—was included. In subsequent models, we substituted HCross for the new metrics one by one, allowing for a comparison between the models with HCross as a predictor and otherwise identical models via the anova function (we report results from the χ^2^-test). We use HCross as the base model because it is a syntactic, entropy-based measure. The metrics by Vanroy and colleagues are also syntactic, but not entropy-based, which can lead to an interesting comparison. If convergence was not possible in subsequent models with the new predictors, we excluded predictors one by one until convergence was possible and compared these to a base model with the same predictors—apart from HCross.

After comparing models with the new predictors to the base model with HCross, we excluded residual outliers (>2.5 SD from the mean). 99.6% of the First Fixation Duration after exclusion of outliers on the basis of the raw data were under 500 ms, while 73% of the excluded outliers were over 500 ms (range 362–1,738 ms). 87.9% of the Total Reading Time after exclusion of outliers on the basis of the raw data were under 5,000 ms. 68.7% of the excluded outliers were over 16,000 ms (range 5,688–164.996 ms). 86.7% of the EKS datapoints after exclusion of the outliers on the basis of the raw data were under 500.000 ms, while 85.8% of the excluded outliers were over 500.000 ms (range 91.580–2.648.372 ms). In other words, extremely long First Fixations, Total Reading Times and EKS were excluded. This is reasonable practice. The fact that often, model results were different after exclusion of residual outliers suggests that these results were often strongly affected by residual outliers, as will be shown. In the interests of transparency, we report model results before and after of exclusion of residual outliers.

Finally, we compared models in which the critical predictors were significant with each other, again via the anova function. We report results from the χ^2^-test, and Akaike's Information Criteria (AIC; Akaike, 1973) and Bayesian Information Criteria (BIC; Schwarz, 1978) are used as indicators of goodness-of-fit of individual models without outliers. We also report marginal *R*^2^ for both versions of each model (with and without residual outliers), which reports the variance of the fixed effects only. In all models, skewness was below |1| and kurtosis below |3| after exclusion of residual outliers. Variance inflation factors in all models were below 2.

## 4. Results

In this section, we present the effects of the predictors word_cross, seq_cross, astred_change, absolute Cross, HCross, HTra, HSTC on three eye-tracking measures: First Fixation Duration, Eye-Key Span, and Total Reading Time on source tokens. In the overview tables, the “ANOVA (HCross)” column compares each model individually with HCross (χ^2^). This HCross model is always shown first. “ANOVA” compares for each model whether it significantly improved over the previous model (models are ordered based on BIC/AIC values with the best fitting model at the bottom). “base” indicates when a model has been used as the first reference model in an ANOVA. When the models are compared, all residual outliers are included. The variance that they account for is given in “*R*^2^ (outliers).” Separate models are also built that exclude for each model its respective residual outliers. These results are reported in “*R*^2^ (no outliers).” In each table only those predictors are included that had a significant effect (with or without outliers) on the dependent variable. Significance of the specific predictor under scrutiny are given in the *p* columns. The individual significance levels of secondary fixed effects (STid, word ID, frequency) were not reported but in all cases they were significant (*p* <0.05). The BIC and AIC columns are given for transparency to indicate the absolute goodness-of-fit of the models (lower is better), as discussed in section 3.3.

### 4.1. First Fixation Duration

Table 1 shows the summary of significant effects on First Fixation Duration (the earliest measure) of which there are few. HCross, word_cross and HSTC have a significant effect. HCross performs best in terms of BIC/AIC as well as *R*^2^ when outliers are included. Neither word_cross nor HSTC perform better according to the ANOVA. However, when outliers are removed, only word_cross has still a significant effect suggesting that outliers were driving the effects in HCross and HSTC in the first place. Only very little variance is explained in these settings.

**Table 1:** Summary of effects on First Fixation Duration (FFDur).

	**w. residual outliers**						**w.o. residual outliers**	
	**ANOVA (HCross)**	**ANOVA**	**BIC**	**AIC**	**p**	**R^2^**	**p**	**R^2^**
HCross	base	base	9154.9	9106.1	0.018^*^	0.0023	0.077	0.0023
HSTC	ns	ns	9156.6	9107.7	0.046^*^	0.0021	0.153	0.0022
word_cross	ns	ns	9156.1	9107.2	0.034^*^	0.0021	0.023^*^	0.0025

* *p < 0.05; ns = not significant*.

*See the introductory paragraph in section 4 for an explanation of the column names*.

**Figure 3:**
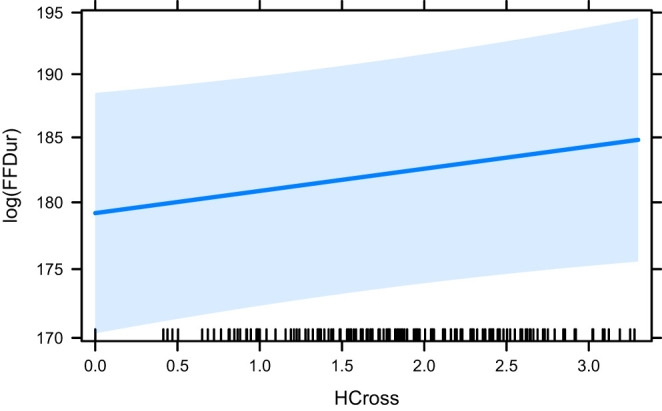
The effect of HCross on the logarithm of FFDur.

**Figure 4:**
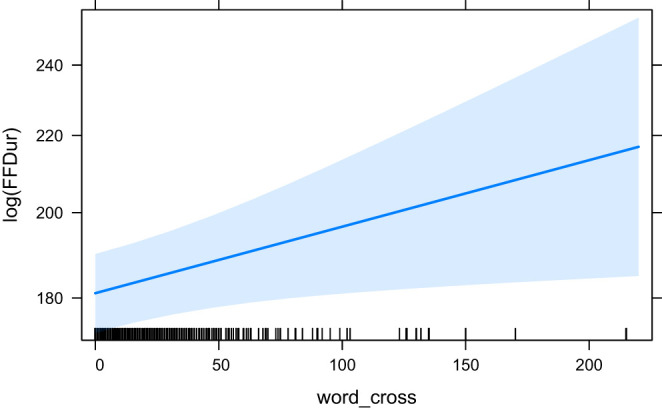
The effect of word_cross on the logarithm of FFDur.

**Figure 5:**
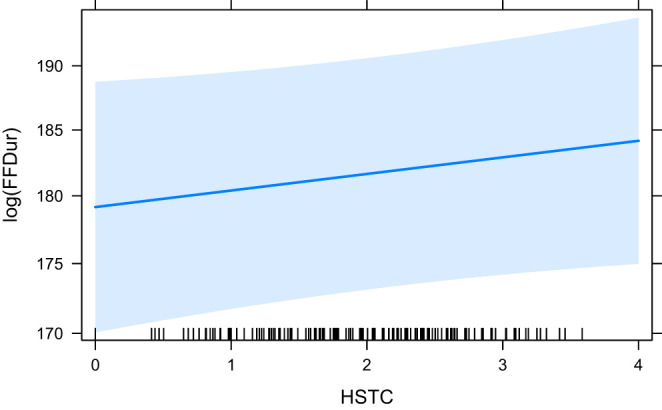
The effect of HSTC on the logarithm of FFDur.

The effect plots for the base model HCross, word_cross and HSTC are given in Figures 3–5, respectively. Important to note is the difference in scale of the y-axis.

### 4.2. Eye-Key Span

Eye-Key Span is considered a late measure, assuming that the translator has fixated a word long enough to at least start producing a translation for it. It does imply, however, that initial problems have been resolved when the production of a word starts (but revision may still happen at a later stage). Many predictors show a significant effect. However, seq_cross only converged when the word ID (the index of the word in the sentence) was excluded as a predictor (the corresponding model is called seq_cross^+^). Therefore, a separate HCross model was built (HCross^+^) that similarly contains the source text ID (the index of the word in the text) and word frequency, but not the word ID. With these fixed effects, seq_cross performs significantly better than HCross according to the ANOVA but it is also evident from their respective BIC/AIC values. On top of that, HCross does not have a significant effect in this context. For that reason, the HCross^+^ model was not included in the second ANOVA. All results with respect to EKS are given in Table 2.

**Table 2:** Summary of effects on Eye-Key Span (EKS).

	**w. residual outliers**						**w.o. residual outliers**	
	**ANOVA (HCross)**	**ANOVA**	**BIC**	**AIC**	**p**	**R^2^**	**p**	**R^2^**
HCross^+^	base				0.146	0.0088	0.515	0.0073
seq_cross ^+^	^***^	base	20259.6	20213.3	0.047^*^	0.0090	0.019^*^	0.0079
HCross	Base	^***^	20200.4	20147.5	0.037^*^	0.0192	0.129	0.0231
abs(Cross)	^***^	^***^	20200.3	20147.4	0.034^*^	0.0190	0.069	0.0230
astred_change	^***^	^***^	20200.2	20147.3	0.032^*^	0.0192	0.040^*^	0.0229
HTra	^***^	^***^	20196.0	20143.1	0.003^**^	0.0204	0.008^**^	0.0240
HSTC	^***^	^***^	20192.4	20139.5	0.000^***^	0.0207	0.002^**^	0.0243

+ *Without word_id as a predictor*.

* *p < 0.05;*

** *p < 0.01;*

*** *p < 0.001*.

*seq_cross only converged without word_id (ID in the sentence)*.

*See the introductory paragraph in section 4 for an explanation of the column names*.

**Figure 6:**
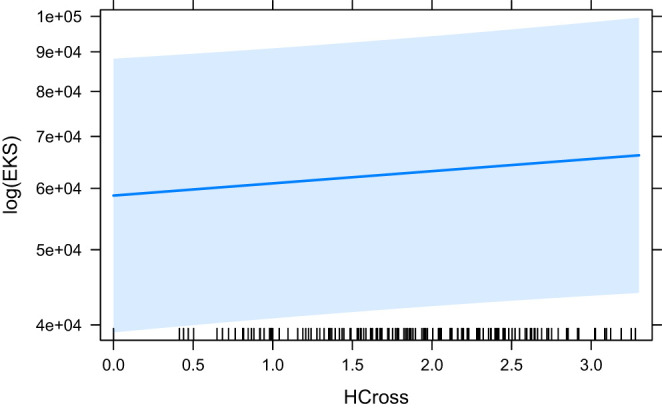
The effect of HCross on the logarithm of EKS.

**Figure 7:**
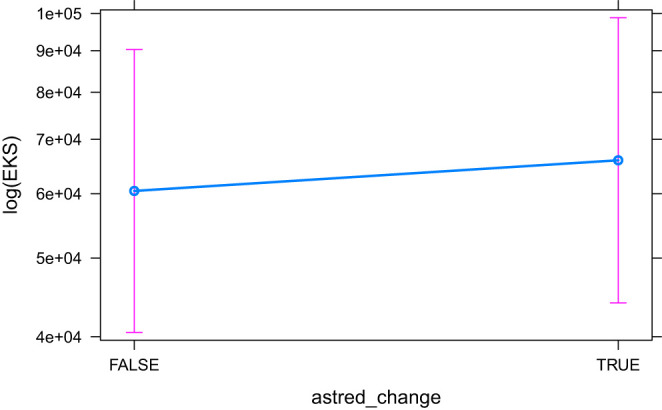
The effect of astred_change on the logarithm of EKS.

**Figure 8:**
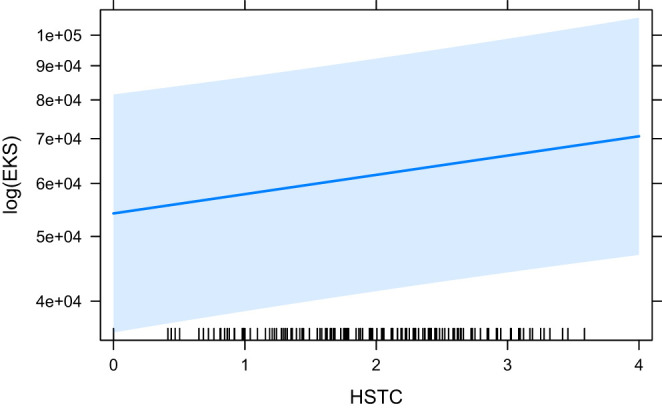
The effect of HSTC on the logarithm of EKS.

The models that did converge with all secondary predictors and that are significant, are HCross, absolute Cross, astred_change, HTra and HSTC. The base model HCross (Figure 6) is significantly outperformed by other predictors and its variant without residual outliers is not significant. The same is true for absolute Cross. astred_change has a significant effect both with and without outliers (Figure 7). Word translation entropy (HTra) and especially HSTC (Figure 8) provide the best fitting models to the data.

### 4.3. Total Reading Time

Similar to Eye-Key Span, Total Reading Time (the latest measure which includes all fixations on a token), is affected by many predictors (Table 3). The base model, HCross, does not have a significant effect so it is no surprise that all other predictors that have a significant effect also perform significantly better than HCross [“ANOVA (HCross)”]. Most predictors have a significant effect with and without residual outliers with the exception of word_cross, which is not significant without. With outliers included in the model it is only marginally significant (*p* = 0.058; in all others cases ^*^p <0.05). seq_cross and HSTC, word group based metrics, are the best performing models according to their BIC/AIC, with HSTC coming out on top. Their effect is highly significant (*p* <0.01). Absolute Cross is the third best fitting model followed by HTra and finally word_cross. The fixed effects in the HTra model explains the most variance in Total Reading Time, however. Note that HCross did not have a significant effect. Therefore, it was not part of the second ANOVA comparison. In that case, the word_cross model was the reference model (because it has the highest BIC/AIC), although it was just marginally significant in the first place.

**Table 3:** Summary of effects on Total Reading Time of source tokens (TrtS).

	**w. residual outliers**						**w.o. residual outliers**	
	**ANOVA (HCross)**	**ANOVA**	**BIC**	**AIC**	**p**	**R^2^**	**p**	**R^2^**
HCross	base				0.235	0.0345	0.362	0.0395
word_cross	^***^	base	22593.4	22537.6	0.058^*^	0.0346	0.062	0.0346
HTra	^***^	^***^	22592.5	22536.6	0.033^*^	0.0358	0.016^*^	0.0417
abs(Cross)	^***^	^***^	22591.1	22535.3	0.015^*^	0.0348	0.004^**^	0.0400
seq_cross	^***^	^***^	22590.1	22534.3	0.009^**^	0.0349	0.005^**^	0.0401
HSTC	^***^	^***^	22589.7	22533.9	0.007^**^	0.0359	0.004^**^	0.0411

* *p < 0.06;*

** *p <0.01;*

*** *p < 0.001*.

*See the introductory paragraph in section 4 for an explanation of the column names*.

*BIC/AIC columns have been rounded for conciseness sake but they are in descending order*.

**Figure 9:**
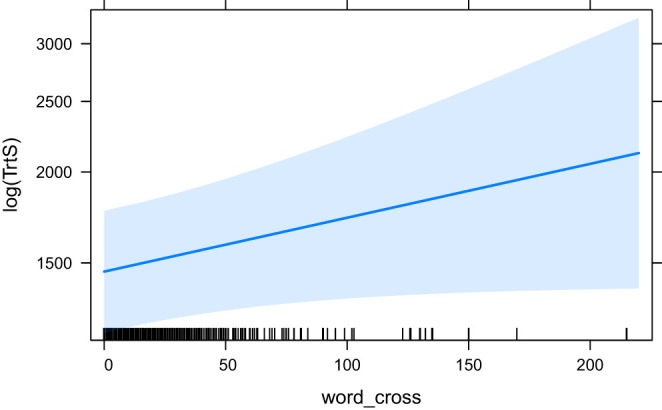
The effect of word_cross (base model) on the logarithm of TrtS.

**Figure 10:**
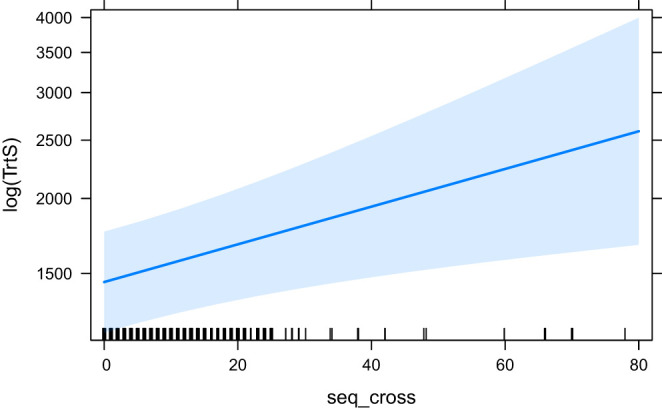
The effect of seq_cross on the logarithm of TrtS.

**Figure 11:**
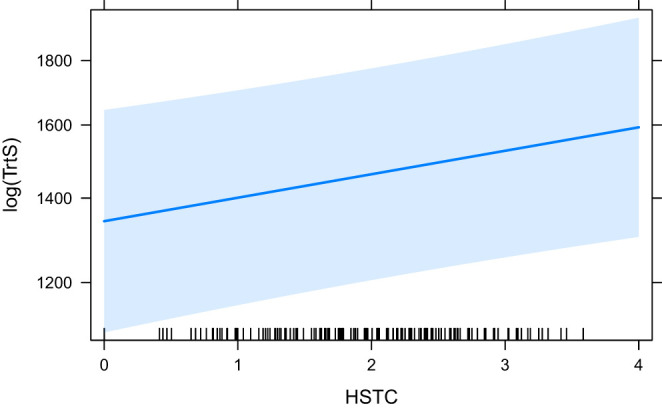
The effect of HSTC on the logarithm of TrtS.

The effects of word_cross (the base model for the ANOVA comparison), seq_cross and HSTC are visualised in Figures 9–11, respectively.

## 5. Discussion

In our experiments, we see very little effect of our predictors on the early measure of First Fixation Duration (FFDur) and those that are significant only explain variance by a minimal amount. Furthermore, both HCross and HSTC (both entropy measures) lose their significance when their residual outliers are removed. The effect of HTra and absolute Cross on FFDur as reported in Schaeffer et al. (2016b) could not be reproduced (but this can likely be attributed to the smaller size of our dataset), although HSTC was significant without outliers, which is interesting because it contains both reordering and translation entropy (of the word group). word_cross was significant both with and without outliers but again, the variance explained was very small.

The effects in later measures are much more prominent. In EKS, a positive effect of seq_cross can be observed but the explained variance is low as is the significance of the predictor. This effect is only present when the word ID predictor is dropped. Because of that, a fair comparison cannot be made with the other predictors by themselves for this dependent variable. Except for word_cross, which is not significant, all other predictors show a positive significant effect. Especially the measures involving semantic information perform well (HSTC, HTra), closely followed by structural changes between ST and TT (astred_change). Absolute Cross is further behind, with a considerable gap in BIC/AIC between astred_change. It is also not significant without the outliers. The same is true for HCross. Therefore, we can cautiously confirm the results reported in Schaeffer and Carl (2017) where HCross was shown to affect EKS positively, although the effect disappears when the residual outliers are removed. With more certainty, we report results in line with Schaeffer and Carl (2017) concerning the significant positive effect of HTra on EKS.

In Total Reading Time, similar effects can be observed with respect to the semantic measures (HTra, HSTC). Interesting, however, is that both absolute Cross and seq_cross perform slightly better than HTra in terms of BIC/AIC although HTra still explains more variance. We can therefore also confirm similar findings by Schaeffer et al. (2016b) concerning the effect of HTra on TrtS. word_cross is only marginally significant and only with its residual outliers included, but seq_cross, on the other hand, is highly significant (*p* <0.001) and performs significantly better than absolute Cross, although the difference in *R*^2^ is minimal. All predictors explain more variance in TrtS than any predictor could in EKS. The reason for this may lie in late, conscious processes. Even after a translation is being generated (EKS is the time from the first fixation on a word until the first keystroke that contributes to its translation), additional fixations on a word may indicate control and revision processes that are active. The implication could be that more divergent source and target structure (in terms of the significant predictors) require longer control and/or revision processes but this needs further investigation. Surprisingly, the significant positive effect of astred_change did not continue in TrtS. This could be related to the aforementioned control processes: syntactic divergent structures may have a significant impact on the problem-solving process right before a translation can be produced (right before the first keystroke of the translation of a word; EKS), but as soon as that problem is resolved, such structural issues are not likely to cause issues during later fixations on the word (i.e., during production or revision).

Because both seq_cross and HSTC involve word groups, it is tempting to attribute their significant effects on late processes, especially TrtS, to a gradual increase of the cognitive unit of translation (from individual words to larger groups in later stages of the translation process). However, because absolute Cross is word-based, the suggestion would be that the unit of translation increases in a compounding manner. In other words: in later stages of the translation process, *both* individual words and (surrounding or involved) word groups are important to the translator. During later processes, a translator may be trying to incorporate or resolve larger units while still taking into account the properties associated with the single word. As mentioned before, a lot of research exists on translation units (e.g., Alves et al., 2010; Immonen and Mäkisalo, 2010; Carl and Kay, 2011; Schaeffer et al., 2016a), and we do not make any conclusive interpretations that confirm or refute any of the suggestions, but we observe that the (possibly changing) unit of translation and its corresponding features may play distinct roles during the time course of the translation process. This is similar in thought to Alves et al. (2010, p. 121): “translators navigate between different linguistic units and levels during translation.” Further research in this direction would be useful. Particularly, interaction effects of word-based and group-based metrics on process data can shed a light on the importance of the properties of the involved translation units during different stages of the translation process. In addition, interaction effects between (lexico)semantic and syntactic properties should also prove interesting, and has already been investigated in some detail by Ruiz and colleagues (Ruiz et al., 2008; Ruíz and Macizo, 2019).

Why we found more effects in late measures (EKS, TrtS) compared to early eye-tracking measures is not easy to explain. One possibility is that our metrics especially model language properties that need conscious decisions. Whereas early measures are often indicative of automatic processes, later measures hint toward conscious decision-making and problem solving, which cannot be resolved automatically (Kiraly, 1995; Bell, 1998). This explanation works for the syntactic measures, where it is conceivable that reordering (Cross, word_cross, seq_cross, HCross, partly HSTC) and insertions and deletions (partly what astred_change models) need more specific attention from the translator. But it does not explain why semantic measures such as HTra and HSTC only have a late effect; the variance in FFDur that is explained by the fixed effects (with HSTC) is very small and HSTC does not have a significant effect when residual outliers are excluded. It may be the case that TL features *are* activated during first contact but that they simply do not pose a problem yet. Another likely explanation is that more data (in terms of the number of data points) is needed to show consistent, early effects.

Conclusions concerning entropy are difficult to make because a variety of factors are involved. HTra and HSTC both have a semantic component, whereas HCross and HSTC contain syntactic information. HSTC involves word groups, whereas HTra and HCross are metrics on the word level. A single statement on the effect of entropy cannot be made. What we can indefinitely say, though, is that more translations could change the picture. Carl (in press) shows that HTra scores only approximate a real population with a Pearson correlation of more than *r* = 0.8 when approximately ten translations are available for a given text (we have between nine and eleven). It is hard to tell then whether entropy-based metrics based on more translations would lead to a greater effect on the process data.

Although strong conclusions are hard to draw because of the size of our dataset, our results indicate that particularly late process measures are affected by the predictors. The reason for this may lie in the conscious processes that occur in such late stages, like problem-solving and revision. In addition, we find that HSTC, an entropy-based metric that incorporates both word group translation and reordering probabilities, is the best-fit predictor across the board. This is perhaps unsurprising, exactly because it entails both syntax and lexicosemantic information while also being based on all available translations. In terms of metrics that are not based on probabilities, absolute Cross has a consistent significant effect in the late measures. seq_cross, which is based on word-group reordering, has a particularly strong significant late effect which poses interesting questions about the cognitive unit of translation and how that unit might change during the translation process.

## 6. Conclusion

In this paper we investigated the effect of a number of predictors that each model different parts of the relationship between a source text and its translation(s). Although our results are promising, “it is dangerous to make sweeping generalisations about translation processes” (Tirkkonen-Condit, 2005, p. 406), particularly because our dataset is limited in size. We encourage other research to confirm or refute our findings with experiments involving different tasks (e.g., sight translation) and datasets (different language pairs, more data points). Furthermore, we wish to emphasise that controlled experiments are necessary if fine-grained linguistic concepts are involved whose effects may not be as clear-cut in empirical corpus-based Translation Studies. In future research we want to particularly focus on more language pairs and see how well the effect of syntactic and semantic divergence generalises to other languages. In addition, we would like investigate additional measures, such as differing part-of-speech tags between source words and their translation, and diverging dependency paths (Nikolaev et al., 2020).

Specifically for the PreDicT project, it is very promising to see that metrics that do not rely on multiple translations also show an effect. Ultimately we wish to predict the difficulty of a given source text, and these results indicate that such singular metrics have predictive power as well. Technically speaking, that is very important: it is much easier to find parallel corpora with one translation than with multiple translation. Such large parallel corpora can be used to train a machine learning model to predict these relevant features (e.g., astred_change) for a given source word, which in turn can be used in a translatability measuring system which predicts difficulties for a given source text without access to a translation.

Our main contributions lie in adapting our previous metrics to the existing arsenal of product-based features that can be calculated on a source word and its translation. The implementation of these metrics has been made available to all as an open-source code base. We also confirmed pre-existing findings by fellow researchers in the field and made our own observations by measuring the effect of a set of predictors on translation process data. And finally, with our results we believe to have added interest to a number of existing research questions that are keen to be investigated, especially involving the (size of) the translation unit, the distinction between (lexico)semantic and syntactic predictors (and their relevance in the time course of the translation process), and whether or not entropy-based measures are a necessity in predicting cognitive effort.

## Data Availability Statement

The dataset (Daems, 2016) analysed for this study can be found in the TPR-DB (https://critt.as.kent.edu/cgi-bin/yawat/tpd.cgi; login: TPRDB, password: tprdb). The files present there do not include all the predictors used in this experiment, because we added those ourselves. Curious readers can still reproduce the dataset, however, by using the provided scripts in our library. With our library (https://github.com/BramVanroy/astred), users can calculate our metrics for any given sentence pair and their alignments. A processing script is also provided that takes as input the tables of any studies that can be downloaded from the TPR-DB and adds our metrics. As such, our metrics can be applied to any existing or new study that is uploaded to the TPR-DB.

## Author Contributions

BV, MS, and LM discussed and agreed upon the experimental design of the study. Data (pre)processing (adding features, annotating data, removing unreliable data points) was done by BV. MS performed all of the statistical experiments in R. The results were discussed and agreed upon by BV, MS, and LM. BV wrote the first draft of the article with the exception of the description of the creation of statistical models, which was written by MS and revised by BV for consistency with the rest of the text. MS and LM made suggestions for improvement, which BV took into consideration in subsequent revisions. All authors contributed to the article and approved the submitted version.

## Conflict of Interest

The authors declare that the research was conducted in the absence of any commercial or financial relationships that could be construed as a potential conflict of interest.

## Publisher's Note

All claims expressed in this article are solely those of the authors and do not necessarily represent those of their affiliated organizations, or those of the publisher, the editors and the reviewers. Any product that may be evaluated in this article, or claim that may be made by its manufacturer, is not guaranteed or endorsed by the publisher.

## Footnotes

^1^ https://research.flw.ugent.be/en/projects/predict

^2^See http://universaldependencies.org/ for label descriptions.

^3^“Label” in the context of tree edit distance refers to any content that is inside a tree's node and not solely to dependency labels (although in regular dependency trees the label is indeed always a dependency label). E.g., in Figure 1, “This:*det*” is a label of the bottom-right node in the tree.

^4^The implementation of the metrics is available on Github https://github.com/BramVanroy/astred.

^5^ https://lexile.com/

^6^P33 was not included in the original ROBOT dataset. The reason for this is not known to us.

^7^ https://sites.google.com/site/centretranslationinnovation/tpr-db

^8^ https://github.com/BramVanroy/astred/blob/master/examples/add_features_tprdb.py
